# Long-Term Outcomes of Osteochondral Allograft with Osteogenic Protein-1 Augmentation: A Twelve-Year Follow-Up

**DOI:** 10.1155/2023/3842443

**Published:** 2023-09-25

**Authors:** Eric Assid, Andrew Renshaw, Mawadah Samad, Richard Tupler, Deryk Jones

**Affiliations:** ^1^Ochsner Health System, New Orleans, LA, USA; ^2^The University of Queensland, Brisbane, Queensland, Australia

## Abstract

**Background:**

Osteochondral lesions (OCLs) can significantly impact functional status and activities of daily living. Weightbearing joints are disproportionately affected due to considerable biomechanical forces in these areas. Various biologic reconstructive procedures such as microfracture, osteochondral autograft transfer (OATS) or allograft transplantation (OCA), and matrix-induced autologous chondrocyte implantation (MACI) are utilized by surgeons to treat OCLs. The integration of osteochondral allografts can restore knee function and maintain the integrity of adjacent joint surfaces. Bone incorporation has been linked to successful outcomes following OCA. Pulse lavage and carbon dioxide have been used to remove marrow elements from the superficial, middle, and deep layers of the allograft; this has been combined with the use of various biologics such as bone marrow aspirate or whole blood to augment donor bone incorporation into the host bone. We present an innovative augmentation approach in OCA transplantation demonstrating excellent incorporation of an osteogenic protein-1 (OP-1) implant (Stryker, Kalamazoo, MI) to treat a large fresh osteoarticular allograft. *Case Presentation*. We present a 51-year-old male who received OCA augmented with an OP-1 implant (Stryker, Kalamazoo, MI) in 2011. Due to subsequent ACL reconstruction for two years and medial meniscal repair four years following OCA transplantation, we were able to arthroscopically evaluate graft status at short- and intermediate-term follow-ups. Positive findings were further verified with radiographic imaging and patient-reported outcome measures (PROMs).

**Conclusion:**

OP-1 implants aided in the bone incorporation of a large osteochondral allograft, restoring a high functional level in a demanding sport.

## 1. Introduction

Osteochondral defects or lesions (OCLs) are estimated to be present in 60% of orthopedic patients who undergo arthroscopy [1]. OCLs in the knee can occur from various mechanisms [2]. Cartilage degeneration may occur from a host of biomechanical factors, such as macro- or microtrauma, as well as biological factors such as osteonecrosis, osteochondritis dissecans, infection, and genetic predisposition [2]. OCLs can be asymptomatic but can also cause painful locking and catching sensations, leading to functional impairment [2]. There is evidence that OCL may propagate further cartilage loss and result in eventual osteoarthritis (OA), even in healthy adults [3]. International Cartilage Repair Society (ICRS) grades have been positively correlated with the development of OA, with some studies suggesting defect size is a contributing factor [2, 4, 5].

Our case presents a 51-year-old male who received OCA augmented with an OP-1 implant (Stryker Co., Kalamazoo, MI), a bone morphogenetic protein (BMP-7), and a type 1 collagen product. The allograft was obtained from AlloSource® (Centennial, CO); large 12 cm^2^ trochlear and 4 cm^2^ medial femoral condyle grafts were transplanted. Both grafts were augmented with OP-1 implants. The trochlear graft was implanted with a shell technique augmented with a titanium screw and chondral dart fixation. The osteochondral allografts transplanted demonstrated greater than 12-year survivorship, excellent graft bone integration, and articular surface maintenance with no adverse side effects. Knee Injury and Osteoarthritis Outcome Scores (KOOS) subscales, the Visual analog scale for pain (VAS), the International Knee Documentation Committee (IKDC), Lysholm, the Physical Health Index (PSF-12), and the Mental Health Index (MSF-12) were collected to verify the functional return. Repeat imaging at early, intermediate, and long-term follow-up was obtained to assess bone incorporation. Two repeat arthroscopic evaluations allowed direct assessment of graft integrity 2 and 4 years following transplantation.

## 2. Case Report

We present a 51-year-old male karate instructor with right knee patellofemoral and medial compartment chondromalacia with multiple loose bodies. The patient failed conservative treatment with viscosupplementation and physical therapy, at which point osteochondral allograft transplantation was performed. Both osteochondral allografts were augmented with OP-1 implants (Stryker Co., Kalamazoo, MI) to aid in graft bone integration and survivorship. Later, direct arthroscopic visualization was performed due to subsequent anterior cruciate ligament (ACL) reconstruction for 2 years and medial meniscal repair for 4 years following OCA treatment. The patient has maintained excellent functional scores greater than 12 years following initial transplantation.

**Figure 1:**
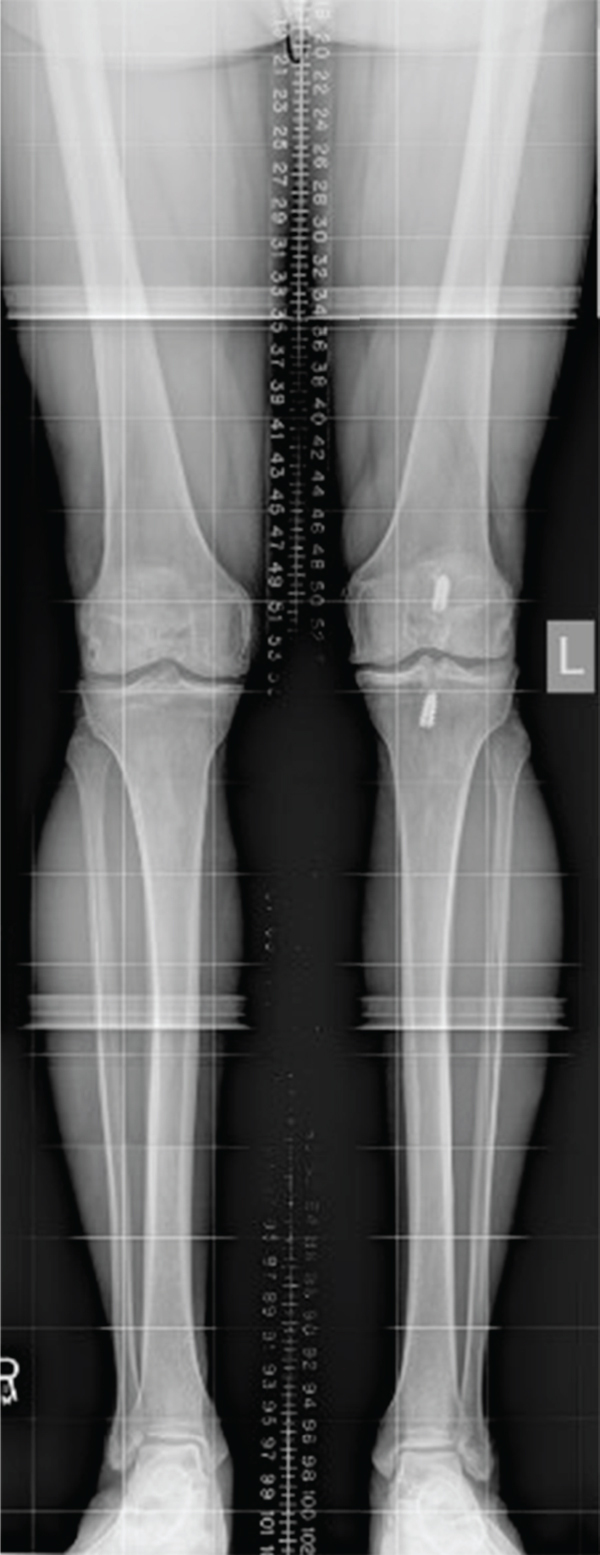
Hip to ankle long-leg standing radiograph demonstrating bilateral symmetric 3° genu valgum.

**Figure 2:**
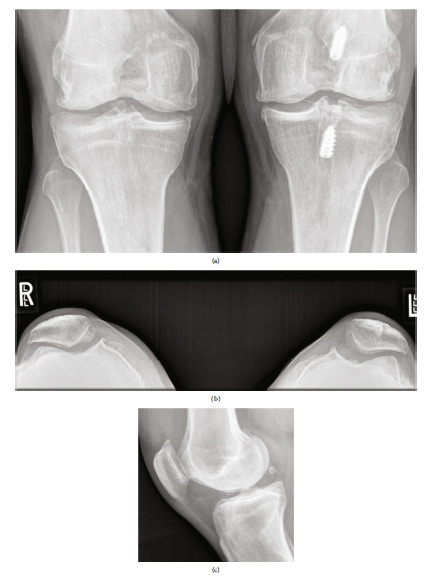
(a–c) Preoperative radiographs. (a) Anterior-posterior standing view with previous ACL reconstruction in the left knee and the Kellgren-Lawrence (KL) grade 2 right and grade 3 left findings. (b) Bilateral Merchant's views demonstrating lateral patellar tilt and mild osteophyte formation. (c) Right knee lateral view.

Our patient initially presented in 2010 with right knee pain reported as three out of ten, characterized by achy stiffness, frequent swelling, weather-related symptoms, and pain at rest. He reported significant functional impairment with difficulty squatting, kneeling, descending stairs, and performing martial arts. The patient had previously been treated with an arthroscopic chondroplasty 3 months prior to the planned OCA treatment. Operative findings were ICRS grade 4 trochlear and medial condyle lesions as well as a grade 2 patellar lesion. Conservative management was pursued initially with viscosupplementation, weight loss, nonsteroidal anti-inflammatory medications (NSAIDs), and formal physical therapy. He was unable to return to full activity; he was a fifth-degree black belt actively participating as a professional karate instructor. The patient reported 8/10 pain on the visual analog pain scale (VAS) with high impact activity; on physical examination, mild valgus alignment was demonstrated bilaterally (Figure 1); right quadriceps atrophy was noted with a persistent small effusion. The right knee demonstrated moderate patellofemoral crepitus, a positive patellar grind, and mild parapatellar pain. Radiographic evaluation with bilateral knee series demonstrated Kellgren-Lawrence- (KL-) 2 changes on the right and KL-3 findings on the left asymptomatic knee with retained screws from prior ACL reconstruction on the left (Figure 2(a)). Radiographic abnormalities were notable in the patellofemoral joint, demonstrating narrowing along the lateral facets bilaterally with mild lateral patellar tilt and subluxation on Merchant's view (Figure 2(b)). The Insall-Salvati ratio was within normal limits with a minor inferior pole patellar tendon enthesopathy on the lateral view of the right knee (Figure 2(c)).

**Figure 3:**
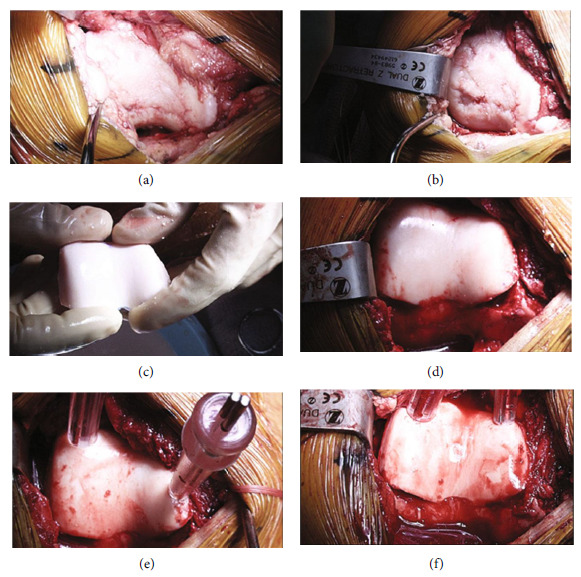
(a, b) Open view 3.0 × 4.0 cm trochlear osteochondral defect. (c) Fresh OCA trochlear bulk allograft 3.0 × 4.0 cm, thickness 5-7 mm contoured. (d) Bulk allograft positioned in defect. (e) Chondral darts placed around the periphery to provide preliminary fixation. (f) Additional titanium 2.0 mm screws placed peripherally on the nonarticular margin of the allograft.

**Figure 4:**
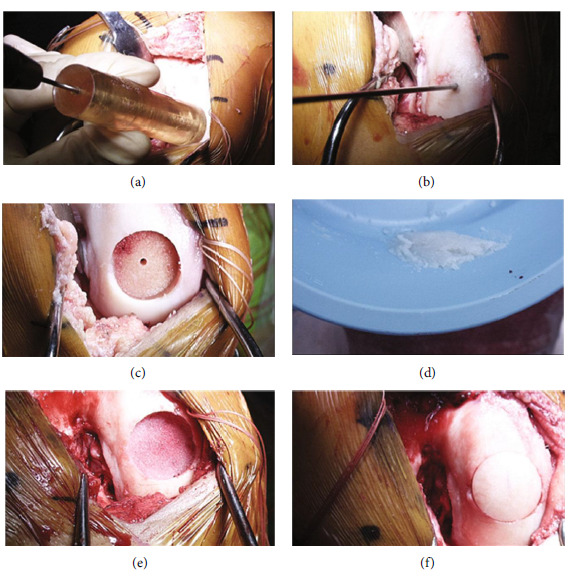
Open view. (a) Lesion gauge used to size medial femoral condyle (MFC) lesion. (b) Guide pin centered in MFC lesion. (c) Reamed MFC lesion, 22.5 × 10 mm in diameter. (d) OP-1 implant preparation with normal saline prior to implantation. (e) OP-1 implant placed at the base of reamed defect. (f) 22.5 × 10 mm dowel graft impacted into position.

**Figure 5:**
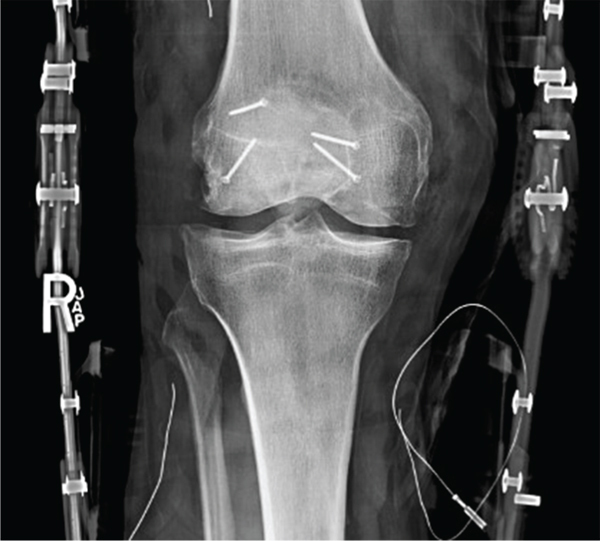
Postoperative AP view demonstrating screw fixation laterally and medially along the trochlear graft and maintained medial/lateral joint spaces after medial femoral condyle graft transplantation.

Due to a failure of conservative measures following arthroscopic chondroplasty with severe patellofemoral findings, the decision was made to proceed with fresh osteochondral allograft transplantation. Arthroscopy was performed, demonstrating ICRS grade 2 changes along the central portion of the patella; grade 4A changes were noted along the articulating trochlea and medial femoral condyle with an intact medial meniscus. The lateral compartment demonstrated an intact lateral meniscus and a 1 cm-diameter ICRS grade 2 lateral femoral condyle lesion which was arthroscopically debrided prior to arthrotomy. Arthroscopic instrumentation was removed from the knee, and a medial subvastus approach was performed with lateral patellar subluxation. Verification of a grade 4A trochlear lesion measuring 3 × 4 cm was made after arthrotomy (Figures 3(a) and 3(b)). The medial compartment demonstrated a 22.5 mm-diameter femoral condyle lesion (Figures 4(a) and 4(b)). The trochlear region was exposed. We then used a 15-blade to demarcate the trochlear lesion itself, extending down to the normal articular cartilage along the periphery of the trochlear region. A sagittal saw was placed along the periphery of the trochlear lesion, and an osteotome elevated the damaged structure directly from the bony bed. We then measured the defect and used the sagittal saw to create an appropriately sized trochlear osteochondral allograft (Figure 3(c)). The trochlear allograft was 3 × 4 cm in dimensions with a bony shell of approximately 5 mm at the periphery and 6 to 7 mm at the central trochlear region. It was contoured appropriately and rounded off from the edges. We then placed this into the trochlear defect to ensure anatomic fit (Figure 3(d)). After confirming the anatomic fit, we saved this graft in saline for later use in the case. The distal femur was exposed, and a central pin was placed into the medial femoral condylar lesion (Figures 4(a) and 4(b)). The lesion was reamed to a diameter of 22.5 mm, a depth of 8 mm at the peripheral edge, 7 mm at the proximal and distal edges, and 4 mm at the intercondylar notch area (Figure 4(c)). Using an Arthrex cutting block, we created a 22.5 mm diameter by 7-8 mm graft. It was contoured and rounded. Prior to the implantation of this dowel graft, we opened a sterile OP-1 implant vial containing recombinant BMP-7 and bovine collagen. The OP-1 implant was mixed with 5 cc of sterile saline and mixed into a suspension (Figure 4(d)). The solution was gently applied to the base of the medial femoral condylar lesion (Figure 4(e)). The dowel graft was treated with pulse lavage to remove its immunogenic potential. We then impacted the dowel graft anatomically into a position (Figure 4(f)). The OP-1 implant was then placed at the base of the trochlear allograft, which was then placed in position. Chondral darts (Arthrex Inc., Naples, FL) were placed around the periphery of the lesion, and four titanium 2.0 modular screws (DePuy Synthes, Warsaw, IN) measuring 40 to 45 mm in length were placed at the four corners of the trochlear graft, stabilizing the trochlear graft in position (Figures 3(d)–3(f)). We added an autologous bone graft saved from site preparation and applied it around the periphery of the trochlear graft (Figure 5). We then exposed the patella and debrided osteophytes from the periphery of the patella. The knee was then allowed to reduce back into the anatomic position with the knee at 30 degrees of flexion, and the drain was in the superolateral aspect of the knee. Standard closure was performed, and the knee was injected with 10 mL of platelet-rich plasma (PRP) prepared with a low white cell count utilizing the Cascade system (MTF Biologics, Edison, NJ). Postoperatively, 25% partial weightbearing was initiated for 2 weeks, followed by progression to full weightbearing over 6 weeks; gait was maintained locked in extension for 6 weeks; a progressive range of motion was allowed as tolerated, limiting open chain exercise for 4 months postoperatively. A full return to karate was allowed at 6 months once CORE and single-leg balance control were demonstrated.

**Figure 6:**
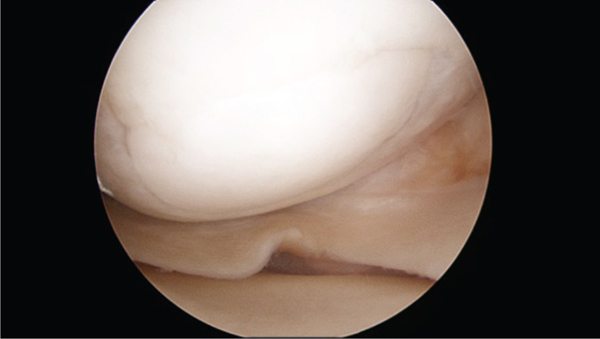
At time of ACL reconstruction 2 years following transplantation of medial 22.5 × 10 mm dowel allograft with maintained articular cartilage surface and excellent incorporation.

**Figure 7:**
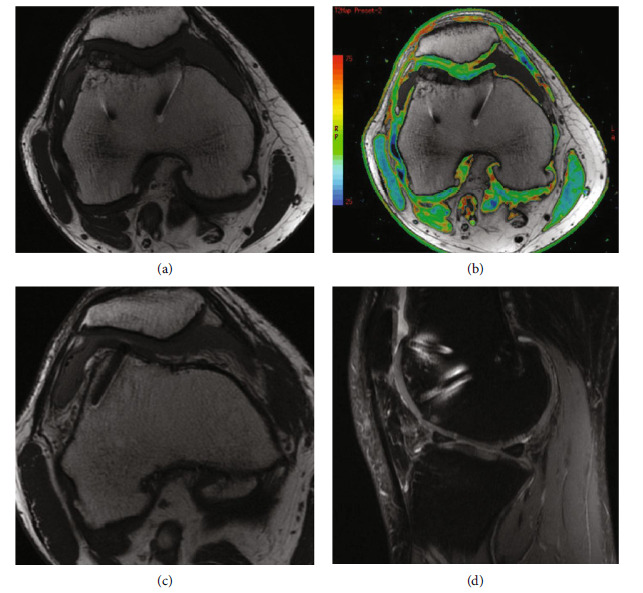
Two-year postoperative MRI. (a) Axial metal artifact reduction sequence (MARS) image demonstrates previous trochlear osteochondral allograft well integrated with intact articular cartilage. (b) T2 mapping axial image with thinning of medial and lateral patellar facets. (c) Additional axial image. (d) Sagittal MARS proton density-fat suppression image demonstrating maintained trochlear implant congruity and bone incorporation; slight thinning at the inferior patellar articular cartilage is demonstrated as well.

The patient tore his anterior cruciate ligament (ACL) in the right knee 2 years following OCA transplantation. Direct arthroscopic visualization of the trochlear (Brittberg 12/12) and medial femoral (Brittberg 11/12) lesions demonstrated excellent incorporation of the grafts and no signs of delamination (Figure 6). Magnetic resonance imaging 2 years postoperatively showed excellent allograft incorporation and intact articular cartilage at the trochlear and medial femoral condylar OCA sites. MOCART 2.0 scores of 80 and 90 were noted, respectively (Figures 7(a)–7(d)). The ACL tear was treated with autologous bone-patellar tendon-bone reconstruction.

**Figure 8:**
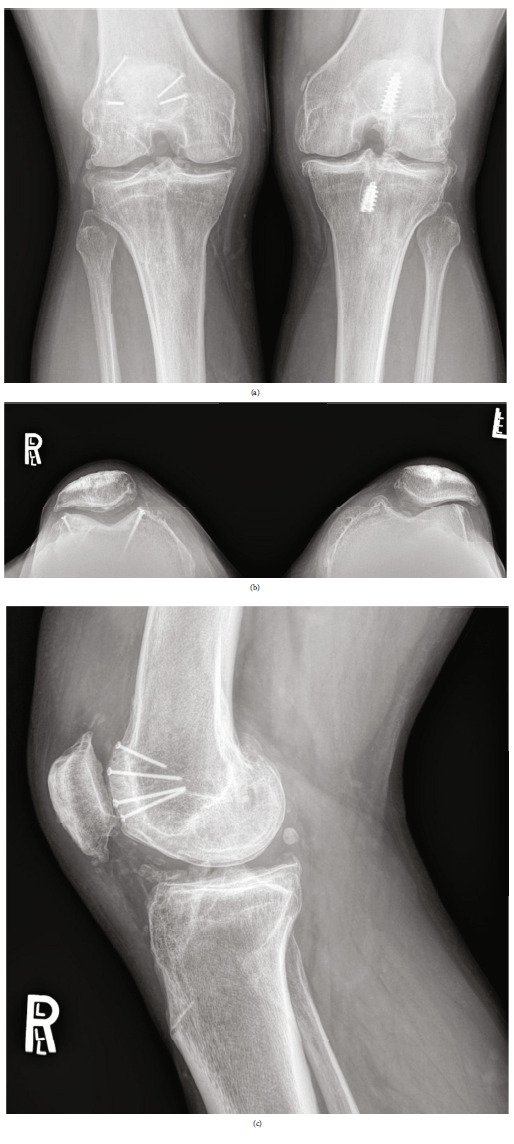
Four-year postoperative radiographs demonstrating excellent bone incorporation and maintained joint spaces with continued KL-2 grade on the right side. (a) Bilateral PA weightbearing. (b) Bilateral Merchant's view. (c) Lateral right knee.

**Figure 9:**
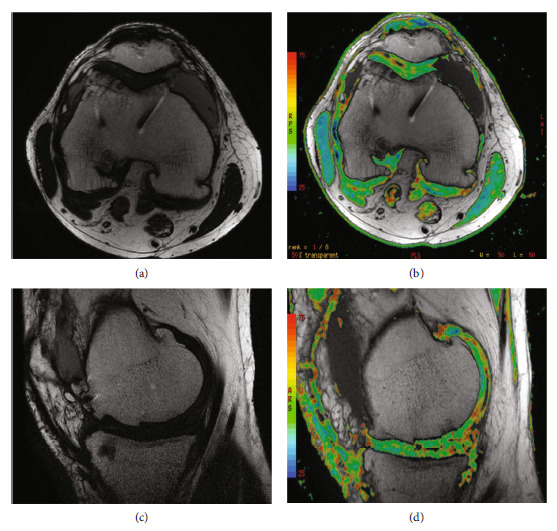
Four-year postoperative MRI. (a) Axial metal artifact reduction sequence (MARS) image demonstrates previous trochlear osteochondral allograft well integrated with intact articular cartilage. (b) T2 mapping axial image with maintained patellofemoral articular cartilage. (c) Sagittal MARS image with well-incorporated medial femoral allograft and preserved articular continuity. (d) Sagittal T2 mapping image demonstrating intact articular cartilage surface at medial femoral condyle allograft.

The patient reinjured his right knee during a 2-day karate tournament in 2016, reporting abnormal soreness and effusion. Radiographs were obtained, demonstrating excellent bone incorporation of the trochlear and medial femoral grafts (Figures 8(a)–8(c)). The examination was consistent with a medial meniscal tear which was verified by MRI preoperatively. This allowed additional imaging of the trochlear and medial femoral OCA grafts, demonstrating excellent bone incorporation of both grafts and maintaining articular surfaces with no signs of delamination 4 years following transplantation (Figures 9(a)–9(d)). MOCART 2.0 scores of the trochlear and medial femoral grafts were 80 and 85, respectively; at arthroscopy, the meniscal repair was performed. Direct visualization was performed of the OCA grafts, demonstrating 12/12 Brittberg scores of the medial femoral condyle and trochlear sites.

**Figure 10:**
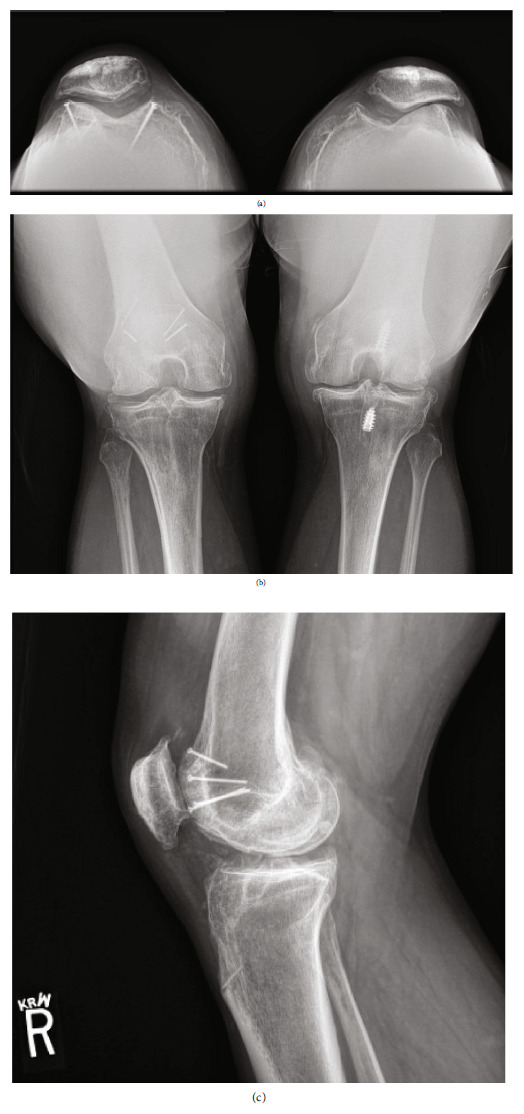
Eight-year postoperative radiographs. (a) Merchant's view demonstrating trochlear implant incorporation with maintained patellofemoral joint space on the right (KL-2) but progression of disease on the left (KL-4). (b)Posterior-anterior Rosenberg view demonstrating maintained medial/lateral joint spaces on the right (KL-2) with left knee progression (KL-4). (c) Right knee lateral radiograph demonstrating excellent trochlear allograft incorporation.

Radiographic evaluation 8 years following OCA transplantation demonstrated excellent bone incorporation of the trochlear and medial femoral grafts; interestingly, the right knee maintained a KL-2 grade, while the left knee demonstrated deterioration to a KL-4 grade (Figures 10(a)–10(c)). Assessment by KOOS subscale scores demonstrated 42-, 54-, 37-, 70-, and 31-point improvements from baseline in pain, symptoms, activities of daily living (ADL), sports, and quality of life (QOL) at 8 years following transplantation; results diminished in pain, symptoms, and ADL (36-, 36-, and 32-point improvements, respectively) 12 years following transplantation, but sports maintained a 70-point improvement, and QOL demonstrated a larger improvement (44-point) 12 years following transplantation. IKDC improved by 48 points, and Lysholm improved by 52 points 8 years following transplantation; once again, improvements diminished slightly in the former (33-point) and the latter (34-point) scores 12 years following transplantation, but these findings clearly met MCID at final follow-up (Tables 1–4).

## 3. Discussion

We present a 51-year-old male Karate instructor who responded favorably to osteochondral allograft transplantation with augmentation using OP-1implant (Stryker Biotech, Kalamazoo, MI) following failed conservative management. The osteogenic protein-1 implant consists of bovine type 1 collagen and bone morphogenetic protein (BMP-7), a subclass of proteins belonging to the transforming growth factor-beta (TGF-b) superfamily [6]. Specifically, BMP-7 has been demonstrated to play a role in the regeneration and repair of cartilage tissue by stimulating the growth and differentiation of chondrocytes, leading to the formation of new cartilage tissue [7, 8]. This protein stimulates stem cells to differentiate into chondrocytes through a process known as chondrogenesis, producing and maintaining the extracellular matrix necessary for healing a cartilage defect [7, 8]. Furthermore, the application of BMP-7 to the affected area has demonstrated anti-inflammatory and analgesic effects through prostaglandin-related pathways, thereby reducing pain and inflammation in the affected area [9]. Other cartilage repair procedures previously mentioned, such as microfracture, mosaicplasty, or matrix-induced autologous chondrocyte implantation (MACI), have the potential to utilize the OP-1 implant to facilitate cartilage repair in the damaged area [6, 10]. The OP-1 implant that was utilized in this procedure was obtained from Stryker Biotech LLC; this company also produced a similarly named OP-1 putty. FDA nonapproval due to questionable marketing practices and adverse effects of the putty has resulted in the removal of OP-1 putty from the market. The product used in this study did not have those same issues, but due to concerns about legal implications with OP-1 putty, OP-1 implant production was discontinued by Stryker as well.

Much contemporary research is ongoing regarding the clinical application of various bone morphogenetic proteins. Perhaps most notable among these is BMP-2, which is currently the only FDA-approved osteoinductive growth factor used as a bone graft substitute. While BMP-7 possesses some similar osteoinductive bioactivities when compared to BMP-2, BMP-7 differs in that it demonstrates anti-inflammatory properties and more chondrogenic activity than BMP-2. The anti-inflammatory effect of BMP-7 has been hypothesized to promote a favorable microenvironment for cartilage healing [9]. In addition, BMP-7's stronger chondrogenic potential in promoting the differentiation of mesenchymal stem cells into chondrocytes offers a potential therapeutic advantage for cartilage restoration procedures [11]. It is within this context that we sought to utilize the favorable bioactivity of BMP-7 as an adjuvant to osteochondral allograft transplantation.

**Figure 11:**
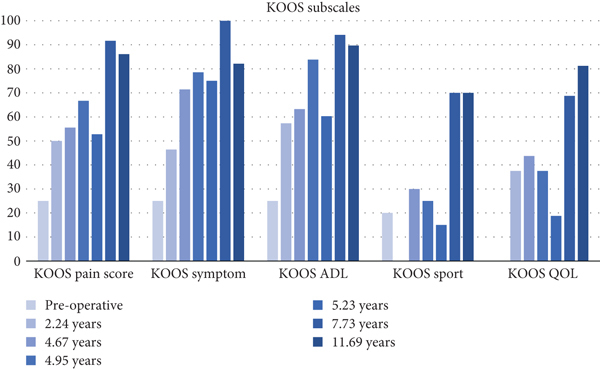
Knee Injury and Osteoarthritis Outcome Scores.

**Figure 12:**
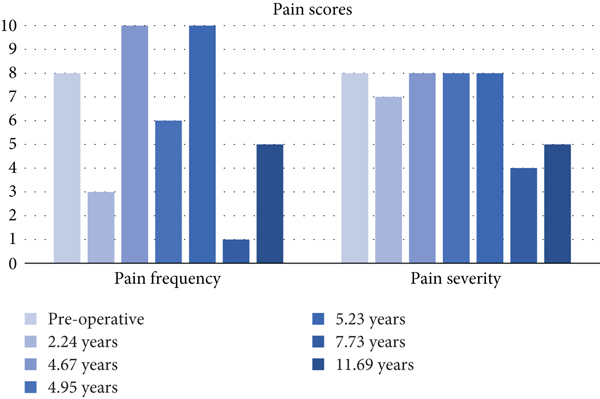
Visual analog scale for pain.

**Figure 13:**
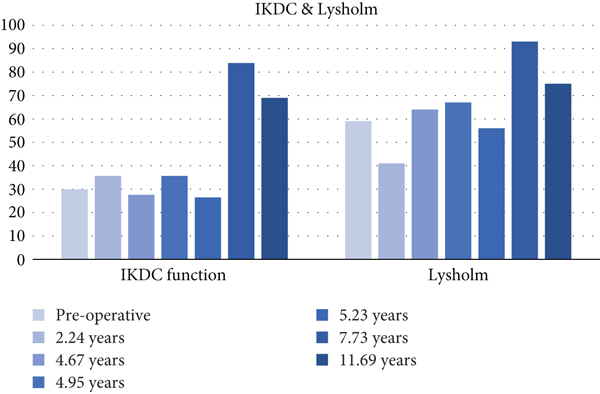
International Knee Documentation Committee and Lysholm scores.

**Figure 14:**
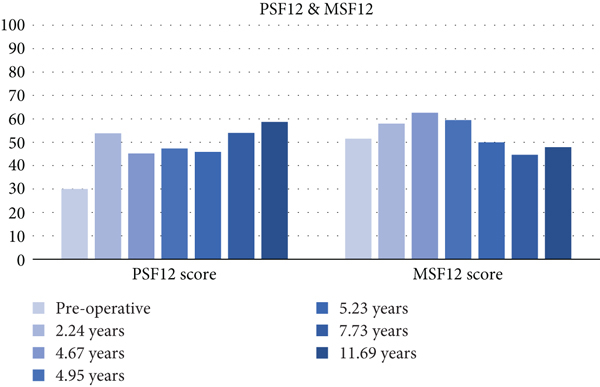
Physical Health Index and Mental Health Index.

The PROMs evaluated in this study were Lysholm, IKDC, KOOS, PSF-12, and MSF-12. Scores closer to 100 represent higher functionality. Our patient showed tremendous improvement in these scores, peaking at 7.73 years postoperatively; these improvements in functionality were maintained four years later. Lower pain scores were also observed during the same period. Our patient preserved the KOOS subscale (Figure 11), VAS (Figure 12), IKDC (Figure 13), Lysholm (Figure 13), PSF-12 (Figure 14), and MSF12 scores (Figure 14). After a thorough review of the medical literature, there was no mention of the usage of an OP-1 implant in cartilage repair procedures with concordant scores that span twelve years.

Treatments for OCLs can be divided into conservative (nonsurgical) and surgical options. Conservative management emphasizing the importance of avoiding repetitive compressive stress on the knee is recommended as first-line therapy [12]. The efficacy of activity restriction (conservative treatment) varies, with radiological healing rates of 10-96% observed in this population [2, 12–14]. The extent of surgical intervention depends on clinical and intraoperative findings. Initial treatment of unstable osteochondral fragments is nonoperative management followed by fixation with absorbable or nonabsorbable implants [2]. A systematic review of thirteen studies that included 148 patients reported radiographic healing outcomes ranging from 67% to 100% with postoperative Lysholm scores ranging from 42 to 98 [15]. However, reoperation rates were reported in up to 44% of patients [2].

The osteochondral allograft procedure that our patient underwent showed radiographic union scores of 86-89% at 2 years [2, 16]. Moreover, 65% showed minimal to no arthritis, with an overall failure rate of 18% [16]. The augmentation of our OCA procedure resulted in favorable outcomes and high satisfaction rates in our patients, suggesting the potential to improve on reported outcomes using large bulk allografts. Other procedures treating chondral damage, such as microfracture, mosaicplasty, and MACI, could potentially benefit from augmentation with BMP-7 as well.

Our case report raises intriguing possibilities, but the generalizability to a larger patient population remains an open question. This case report alone is not sufficient evidence to elicit changes in clinical practice guidelines. The safety of the use of BMP-7 in all types of cartilage procedures and in all types of patients is a legitimate concern. Placement of a large fresh osteoarticular allograft in a high-demand athlete expected to place extreme forces across the patellofemoral joint postoperatively warranted augmentation, guaranteeing full and relatively quick bone incorporation. As a result of further injury to this patient's knee, we were able to repeatedly continue extensive longitudinal follow-up supported by frequent responses to the PROM questionnaires and frequent radiological imaging; this allowed us to detect changes or trends in scores. After an extensive review of the literature, the novelty and educational value that our case presents may provide the groundwork for similar BMP-7 applications and research within other cartilage repair procedures.

## 4. Conclusion

We report on a patient treated with fresh osteochondral allograft transplantation augmented with OP-1 implants following failed conservative treatment. Twelve years following the initial OCA transplantation, the patient demonstrated excellent function and radiographic stability following continued high demand on his knee.

## Figures and Tables

**Table 1:** Knee Injury and Osteoarthritis Outcome Scores.

Postoperative period	KOOS pain score	KOOS symptom	KOOS ADL	KOOS sport	KOOS QOL
Preoperative	25	25	25	20	0
2.24 years	50	46.43	57.35	0	37.5
4.67 years	55.56	71.43	63.24	30	43.75
4.95 years	66.67	78.57	83.82	25	37.5
5.23 years	52.78	75	60.29	15	18.75
7.73 years	91.67	100	94.12	70	68.75
11.69 years	86.11	82.14	89.71	70	81.25

**Table 2:** Visual analog scale for pain scores.

Postoperative period	Pain frequency	Pain severity
Preoperative	8	8
2.2 years	3	7
4.67 years	10	8
4.95 years	6	8
5.23 years	10	8
7.73 years	1	4
11.69 years	5	5

**Table 3:** International Knee Documentation Committee and Lysholm scores.

Postoperative period	IKDC function	Lysholm
Preoperative	29.9	59
2.24 years	35.63	41
4.67 years	27.58	64
4.95 years	35.63	67
5.23 years	26.43	56
7.73 years	83.9	93
11.69 years	68.96	75

**Table 4:** Physical Health Index and Mental Health Index scores.

Postoperative period	PSF12 score	MSF12 score
Preoperative	30.03	51.47
2.24 years	53.8	57.92
4.67 years	45.17	62.56
4.95 years	47.29	59.42
5.23 years	45.85	49.89
7.73 years	53.94	44.62
11.69 years	58.66	47.86

## Data Availability

The data is available on request from the research fellow and/or manager of clinical research and/or clinical research coordinator at Ochsner Hospital for Orthopedics & Sports Medicine.
